# Effects of bulevirtide on atherosclerosis in an ApoE-deficient mouse model

**DOI:** 10.1371/journal.pone.0349211

**Published:** 2026-05-26

**Authors:** Jonas Rusnak, Victoria Delcheva, Dirk Theile, Antje Blank, Norbert Frey, Michael R. Preusch

**Affiliations:** 1 Department of Cardiology, Angiology and Pneumology, Heidelberg University, Medical Faculty Heidelberg/Heidelberg University Hospital Internal Medicine III, Heidelberg, Germany; 2 DZHK (German Centre for Cardiovascular Research), Partner Site Heidelberg/Mannheim, Heidelberg, Germany; 3 Department of Clinical Pharmacology and Pharmacoepidemiology, Heidelberg University, Medical Faculty Heidelberg/Heidelberg University Hospital Internal Medicine IX, Heidelberg, Germany; 4 DZIF (German Center for Infection Research), Partner Site Heidelberg, Heidelberg, Germany; Charite Universitatsmedizin Berlin, GERMANY

## Abstract

**Objective:**

Bile acids are known to have a positive impact on atherosclerosis. The study investigates the impact of sodium taurocholate co-transporting polypeptide (NTCP) inhibition with bulevirtide, organic anion transporting polypeptide (OATP) inhibition with rifampicin, and their combination on bile acid levels and atherosclerosis in apolipoprotein E–deficient (ApoE⁻/⁻) mice.

**Methods:**

Fifty-six female ApoE⁻/⁻ mice on a Western type diet were treated daily for four weeks with bulevirtide (5 mg/kg), rifampicin (20 mg/kg), a combination of both, or vehicle. Plasma bile acids and lipids were measured at sacrifice. Atherosclerotic lesion size was quantified in the aortic sinus, and macrophage content was analyzed by Mac-2 immunohistochemistry.

**Results:**

Plasma cholesterol, low-density lipoprotein (LDL), high-density lipoprotein (HDL), and triglyceride levels remained unchanged across all groups, being accompanied with no changes of atherosclerotic lesion size (p = 0.896). However, treatment with bulevirtide (p = 0.017), rifampicin (p = 0.003), but mostly their combination (p < 0.001) significantly increased plasma bile acid concentrations and altered the macrophage-to-lesion area ratio, at least for the combination therapy group (p = 0.020). The combined treatment exhibited the highest bile acid levels, indicating additive effects of dual transporter inhibition.

**Conclusions:**

While lesion sizes remained unaffected, the combined NTCP and OATP inhibition significantly enhanced bile acid levels and reduced macrophage content in early atherosclerotic lesions of ApoE⁻/⁻ mice. These results highlight the association of bile acid with the modulation of plaque composition as a potential therapeutic mode of action.

## Introduction

Atherosclerosis is a chronic inflammatory disease of the arterial wall and the leading cause of cardiovascular events such as myocardial infarction. In addition to established lipid-lowering therapies, there is growing interest in targeting inflammatory and metabolic pathways to prevent disease progression or stabilize existing plaques [1].

Bulevirtide (formerly known as myrcludex B) is a synthetic peptide developed as an entry inhibitor for hepatitis B and D viruses [2–5]. Its mode of action is based on the competitive inhibition of the sodium taurocholate co-transporting polypeptide (NTCP), a key physiological hepatic transporter for conjugated bile acids and the relevant cellular virus entry receptor [6–9]. Bulevirtide, marketed under the name of Hepcludex and approved by the European Medicines Agency in 2020, has been shown to be a safe and highly effective drug to treat patients with chronic hepatitis B and D, by reducing viral load and normalizing liver enzymes [3,10–12]. By blocking NTCP, bulevirtide also increases circulating bile acid levels in humans, particularly conjugated species, without causing clinical signs of cholestasis [6].

Beyond their role in dietary fat digestion, bile acids act as signaling molecules via nuclear receptors such as the farnesoid X receptor (FXR) and membrane-bound receptors such as Takeda G protein–coupled bile acid receptor 5 (TGR5) [13,14]. Activation of these pathways affects lipid and glucose metabolism, inflammation, and vascular tone [14–17]. Preclinical studies have demonstrated that FXR and TGR5 activation can reduce macrophage-driven inflammation as well as macrophage cholesterol uptake and can prevent atherosclerotic plaque formation [18–22]. In contrast, studies in FXR-deficient (FXR-/-) mice yielded opposing results with regard to atherosclerotic plaque formation [20,23–25]. Accordingly, bulevirtide-mediated enhancement of bile acids should result in favorable vascular effects.

In addition to bulevirtide, rifampicin (a broad-spectrum antibiotic – in the U.S. also known as rifampin) is an inhibitor of organic anion transporting polypeptides (OATPs) and consequently elevates circulating bile acid levels, potentially influencing similar signaling pathways by reducing hepatic uptake of endogenous ligands [26,27]. However, it remains unclear to what extent pharmacologically induced alterations in bile acid homeostasis affect atherogenesis.

Accordingly, the aim of this study was to evaluate the effects of bulevirtide on the extent and composition of atherosclerotic lesions in an apolipoprotein E–deficient mouse model. To determine whether these effects are directly mediated or secondary to elevated bile acid levels, a rifampicin control group was included.

## Materials and methods

### Animals and drug administration

A total of 56 female apoE^-/-^ mice (B6.129P2-Apoe^tm1Unc/J^) were obtained from Charles River Laboratories. Female ApoE ⁻ / ⁻ mice were used because they develop more pronounced atherosclerotic lesions than males, enabling improved detection of treatment effects while avoiding sex-related variability [28–30]. In addition, females exhibit distinct bile acid metabolism, which may enhance the responsiveness to bile acid–targeting interventions such as bulevirtide [31]. The mice were maintained on a Western-type diet (Altromin International, Lage, Germany). By ten weeks of age, these apoE-/- mice begin to develop atherosclerotic lesions in the aortic sinus [32]. This mice model of early-stage atherosclerotic lesions was deliberately selected over an advanced disease model to avoid potential confounding effects associated with irreversible plaque remodeling present at later stages. At this time point, animals were assigned to one of four treatment groups: bulevirtide (5 mg/kg body weight; n = 15), rifampicin (20 mg/kg body weight; n = 15), a combination of bulevirtide and rifampicin at the aforementioned doses (n = 15), or control (0.9% NaCl; n = 11). Treatments were administered once daily via intraperitoneal injection. All animal procedures were approved by the Federal Animal Care and Use Committee of the Regierungspraesidium Karlsruhe under the reference number 35-9185/G-99/17.

Bulevirtide was received from a batch manufactured for a clinical trial (EudraCT: 2017-003137-28) and was a lyophilized powder in vials with 5 mg each. The powder had to be diluted in 1 mL of water for injection within two hours prior to administration and was further diluted to the required dose. The investigational medical product (IMP) was stored at −20 °C.

### Animal sacrifice and tissue collection

Following four weeks of treatment (at 14 weeks of age), mice were euthanized under deep anesthesia (ketamine/xylazine, intraperitoneally) followed by exsanguination via the inferior vena cava for blood collection. Perfusion was carried out through the left ventricle using 10 mL phosphate-buffered saline (PBS), followed by dissection of the thoracoabdominal aorta. Subsequently, a second perfusion with 4% buffered formalin was performed for fixation of the aortic root. After 24 hours, the aortic sinuses were washed again with PBS and incubated in sucrose/PBS solution for 12 hours. Finally, aortic roots were embedded in the water-based fixation compound Tissue-TEK/O.C.T. (Sakura Finetek Europe, Umbach, Germany). Serial sections (5 μm) were then prepared for histological analysis.

A total of 56 mice were used and subsequently euthanized for analysis of the aortic sinus. Animals were housed in the animal care facility at the University of Heidelberg under controlled conditions, including a temperature-regulated environment and a 12-hour light/dark cycle. Trained personnel monitored the animals for any abnormalities twice daily. Humane endpoints were defined as weight loss exceeding 20%, signs of manifest infection (delayed or absent food intake for more than 48 hours), or deterioration in general condition such as apathy. Behavioural abnormalities, including persistent apathy, as well as skin or fur changes with redness and signs of inflammation were also considered humane endpoints. As soon as any of these criteria were met, animals were euthanized. All personnel involved in animal handling and treatment were specifically trained and possessed the requisite qualifications. All animal welfare considerations were strictly observed, including continuous efforts to minimize suffering and distress, the appropriate use of analgesics or anesthetics, and, where necessary, the provision of special housing conditions.

### Determination of plasma lipid concentration

To determine the concentrations of total cholesterol, high-density lipoprotein (HDL), low-density lipoprotein (LDL), and triglycerides, plasma from one-third of the animals in each experimental group was analysed. Triglycerides, total plasma cholesterol and LDL levels were enzymatically determined (CHOD-PAP, Siemens Healthcare Diagnostics GmbH, Eschborn, Germany) at the time of sacrifice.

### Determination of plasma bile acid concentration

To determine the concentrations of bile acids (cholic acid, chenodeoxycholic acids, and their dehydroxylated and conjugated forms), the Bile Acid Assay Kit from Sigma-Aldrich (Taufkirchen, Germany) was used according to the manufacturer’s instructions. Briefly, the bile acids are oxidized by 3α-hydroxysteroid dehydrogenase (contained in the kit), converting NAD to NADH, which in turn reduces a probe to a highly fluorescent product (excitation 530 nM; emission 585 nm).

### Evaluation of lesion size

**Fig 1:**
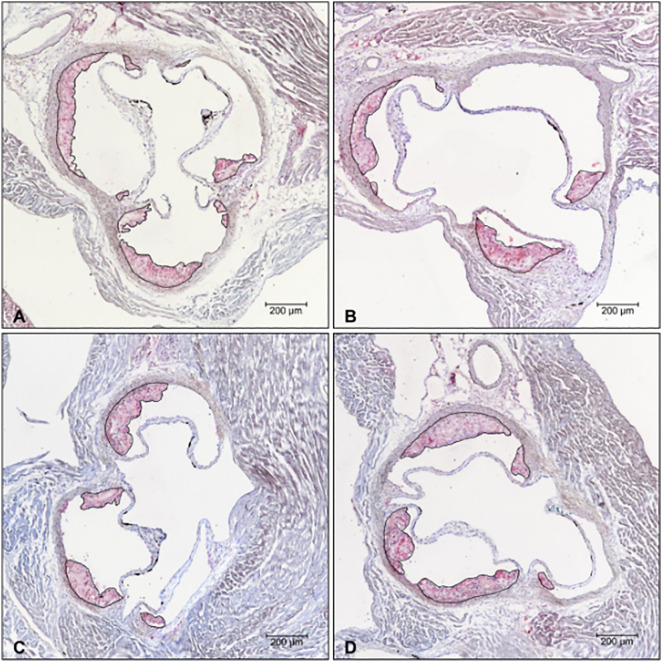
Oil Red O staining – comparison of the four groups. Representative sections from the valvular plane at 4-times magnification. Atherosclerotic lesions are outlined in black. A: Bulevirtide, B: Bulevirtide+ Rifampicin, C: Rifampicin, D: Control.

For the evaluation of lesion size, a total of 50 animals were included in the analysis. Lesion size was investigated in the area of the aortic sinus. Non-axial sectioning of the aortic root or damaging during cross sectioning occurred in six animals. For this reason, one to two animals per group had to be excluded from the analyses. To ensure representative assessment of atherosclerotic lesions, an average of 10 sections per animal were analyzed for lesion size in the aortic root. Oil Red O staining was used as a complementary method for the visualization and quantification of atherosclerotic lesions (Fig 1). The lipid-soluble dye Oil Red O (Sigma-Aldrich, St. Louis, USA) stains neutral lipids red, thereby highlighting lipid deposits in tissue samples. In addition to Oil Red O, hematoxylin was used to stain nuclear components blue. This nuclear counterstaining enhances the contrast between cell structures and lipid deposits, improving the visualization and characterization of atherosclerotic lesions. Oil Red O staining was performed on every second slide per animal. After drying, the sections were mounted using an aqueous mounting medium. Subsequently, the area of the atherosclerotic lesions was measured using the image analysis software ImageJ (Image J, Bethesda, USA).

### MAC-2 Immunohistochemistry

**Fig 2:**
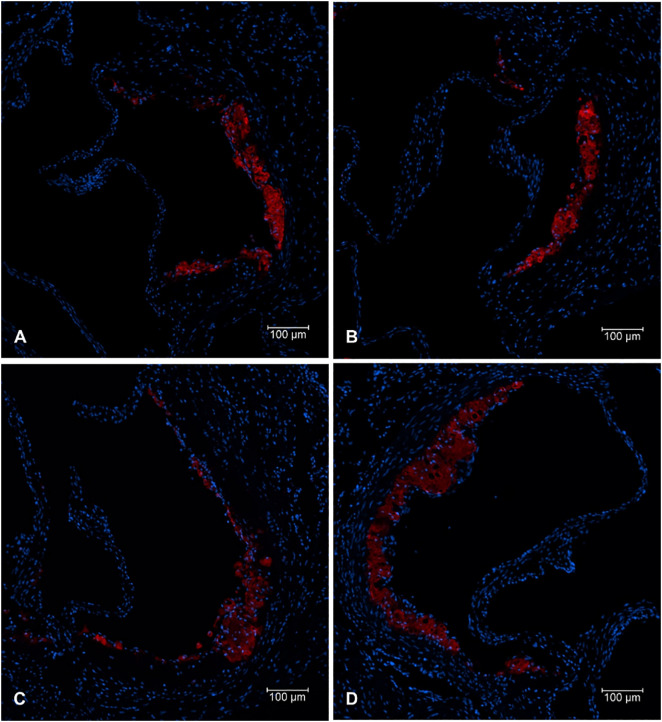
Mac-2 immunofluorescence staining – comparison of the four groups. Representative sections from the valvular plane at 10-times magnification. Mac-2–positive areas are shown in red; DAPI nuclear staining is shown in blue. A: Bulevirtide, B: Bulevirtide + Rifampicin, C: Rifampicin, D: Control.

To analyse the macrophage content within the atherosclerotic lesion, 48 mice were evaluated as unsuccessful staining resulted in an insufficient number of evaluable sections in two additional animals. Mac-2 immunofluorescence staining was performed on one tissue section from every fourth slide per animal (Fig 2). For evaluation of macrophage infiltration, an average of 5 sections per animal were analyzed. Tissue sections of the aortic sinus, located adjacent to the regions with maximal lesion area, were dewaxed and rehydrated. To block endogenous peroxidase activity, sections were incubated in a blocking solution containing bovine serum albumin and PBS (Carl Roth GmbH, Karlsruhe, Germany). Macrophages were detected using a monoclonal rat anti-mouse Mac-2 antibody (Accurate Chemical, New York, USA). The primary antibody was applied overnight at 4 °C in a humidified chamber. Following incubation, the sections were washed three times with PBS and then incubated with biotinylated secondary antibodies (Dianova, Hamburg, Deutschland) for 60 minutes, followed by three washes with PBS.

### Statistical analysis

Data distribution was assessed using the Shapiro–Wilk test. Depending on the distribution, either parametric or non-parametric statistical tests were applied. For normally distributed data, group comparisons were performed using one-way analysis of variance (ANOVA), followed by post hoc testing using Tukey’s honest significant difference method. In cases where the assumption of normality was not met, the Kruskal–Wallis test was used for group comparisons, with Dunn’s test and Bonferroni correction applied for post hoc analysis. Descriptive statistics were calculated for each treatment group. Data are expressed as mean ± standard deviation (SD) for parametric analyses or as median and interquartile range (IQR) for non-parametric analyses. A p-value < 0.05 was considered statistically significant. As this was an exploratory analysis, no adjustments for multiple testing were performed. Statistical analyses were done using RStudio (Posit PBC, Boston, Massachusetts, USA).

## Results

### Animals

The experimental period spanned four weeks. During this time, one animal in the bulevirtide/rifampicin group died prior to meeting the predefined humane endpoint criteria for euthanasia. All remaining animals were euthanized according to protocol for subsequent preparation and analysis of the aortic sinus.

### Effect on plasma lipid level and body weight

For the evaluation of lipid serum concentration 19 mice were included. Serum lipid measurements revealed no significant differences in total cholesterol, LDL cholesterol, HDL cholesterol, or triglyceride levels among the groups (Table 1). In contrast, body weight significantly differed between the groups (p < 0.001). In detail, mice treated with bulevirtide or the combination therapy of bulevirtide/rifampicin displayed significantly lower body weights than control animals. Furthermore, rifampicin-treated animals showed significantly higher body weights compared to the bulevirtide group (S1 Fig).

**Table 1:** Laboratory values.

Variable	Control (n = 4)	Bulevritide (n = 5)	Rifampicin (n = 5)	Bulevirtide + Rifampicin (n = 5)	p-value
Total Cholesterol [mg/dl]	656 (± 107)	760 (± 228)	768 (± 84)	824 (± 96)	0.578
LDL Cholesterol[mg/dl]	630 (± 108)	716 (± 232)	752 (± 88)	804 (± 96)	0.477
HDL Cholesterol [mg/dl]	6 (± 4)	8 (± 4)	4 (± 4)	8 (± 0)	0.514
Triglycerides [mg/dl]	81 (± 15)	98 (± 43)	76 (± 16)	72 (± 22)	0.459

Values of triglycerides are expressed as mean ± standard deviation (SD).

Values of cholesterol, LDL and HDL levels are expressed as median ± interquartile range (IQR).

### Atherosclerotic lesion area and macrophage content

**Fig 3:**
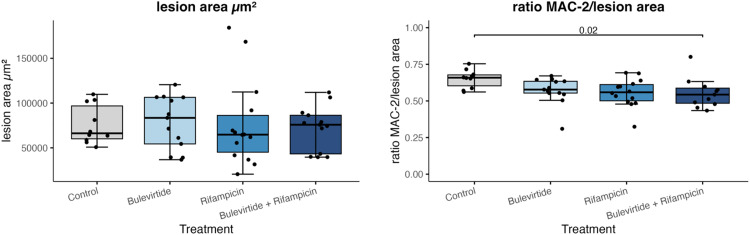
Atherosclerotic lesion area after four weeks of treatment. Data are presented as median ± interquartile range (IQR). Control (n = 10), Bulevirtide (n = 13), Rifampicin (n = 14), combination therapy Bulevirtide/Rifampicin (n = 13).

As illustrated in Fig 3 and Table 2, after four weeks of treatment analyses of the atherosclerotic lesion area revealed no statistically significant differences between the groups (p = 0.896). However, significant differences among the groups were seen regarding the macrophage content of the atherosclerotic plaque represented by the MAC-2/lesion area ratio (p = 0.019). Post hoc analyses revealed that this is mostly related to a statistically significant reduction of the MAC-2/lesion area ratio in the combination therapy bulevirtide/rifampicin group compared to controls (p = 0.020). The groups of mice treated with bulevirtide or rifampicin alone showed solely a numerical reduction in macrophage content compared to the control group. However, these data did not reach statistical significance.

**Table 2:** Lesion area and MAC-2.

Treatment	Lesion Area (μm²) Median (± IQR)	p-value	MAC-2/ Lesion Area Median (± IQR)	p-value
Control	66.220 (36.747)	0.896	0.659 (0.075)	**0.019**
Bulevirtide	83.479 (52.025)		0.578 (0.080)	
Rifampicin	64.743 (41.099)		0.559 (0.111)	
Bulevirtide + Rifampicin	75.815 (43.110)		0.543 (0.103)	

IQR – interquartile range.

Control (n = 10), Bulevirtide (n = 13), Rifampicin (n = 14), combination therapy Bulevirtide/Rifampicin (n = 13 for lesion area; n = 11 for MAC-2/lesion area).

### Bile acid concentration

**Fig 4:**
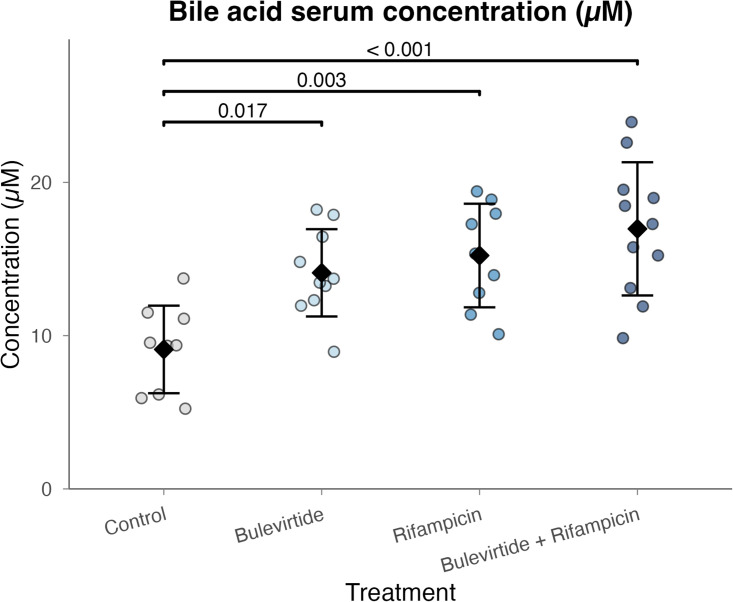
Plasma bile acid concentrations in mice. Data are presented as mean ± standard deviation (SD). Control (n = 9), Bulevirtide (n = 10), Rifampicin (n = 9), combination therapy Bulevirtide/Rifampicin (n = 11).

Effect of the different treatment options were evaluated on their effect on total bile acid concentrations (Fig 4). Total bile acid concentrations were significantly different between the groups (p < 0.001). Specifically, bile acid levels were significantly higher in the bulevirtide group (14.1 ± 2.8 μM; p = 0.017), the rifampicin group (15.2 ± 3.4; p = 0.003), and the bulevirtide/rifampicin combination group (17 ± 4.3 μM; p < 0.001) relative to controls (9.1 ± 2.8 μM). While the combination therapy group showed the highest mean bile acid concentrations among all groups, indicating a potential trend toward additive or synergistic effects, these differences were not statistically significant in the post hoc comparisons versus the monotherapy groups (bulevirtide vs. combination therapy: p = 0.247; rifampicin vs. combination therapy: p = 0.680).

## Discussion

The present study evaluated the effects of bulevirtide, an NTCP inhibitor, on the extent and composition of atherosclerotic lesions in ApoE-deficient mice. To distinguish whether observed effects were directly attributable to bulevirtide or secondary to elevated bile acid levels, rifampicin-treated control groups were included. While neither bulevirtide nor rifampicin monotherapy resulted in changes in macrophage content or lesion size compared to controls, the combination therapy significantly reduced macrophage content within early atherosclerotic plaques. Given that bile acid levels were highest in the combination group, these findings suggest that increased bile acid levels are associated with changes in plaque composition. Thus, short-term modulation of hepatic bile acid transporters and downstream elevations of bile acids appear to predominantly influence plaque composition, with potential effects on lesion size possibly requiring longer treatment durations.

These findings are in line with clinical data showing that both bulevirtide and rifampicin increase plasma bile acid levels in humans [6,26,33]. In addition, experimental studies in mice have demonstrated that NTCP inhibition by bulevirtide raises systemic bile acid concentrations, although compensatory OATP-mediated transport may attenuate the extent of this increase [34,35]. Donkers et al. reported that the full effect of bulevirtide on plasma bile acids becomes only evident in OATP-deficient mice, in which the compensatory function of OATPs is absent [36,37]. Rifampicin, a potent OATP inhibitor in both mice and humans, has likewise been reported to increase plasma bile acids in both clinical and preclinical studies, which again is in line with our results [26,27,33]. Accordingly, the combined treatment group exhibited numerically the highest bile acid concentrations, most likely reflecting the loss of the compensatory OATP effect. The elevated bile acid concentrations may have underscored the observed reduction in macrophage content within atherosclerotic lesions as it is known that lower levels of bile acid concentration are predictive of coronary artery disease in humans [38].

Although elevated bile acid levels in the groups treated with bulevirtide and/or rifampicin could be expected to influence lesion size, no significant differences in lesion area were observed in the present study. This stands in contrast to a recently published study by *Porteiro et al.*, in which NTCP inhibition with bulevirtide in OATP1a1⁻/⁻ Ldlr⁻/⁻ mice indeed resulted in a significant reduction in atherosclerotic lesion size [39]. However, this effect could not be found in the group of Ldlr⁻/⁻ mice with preserved OATP function, suggesting that both transporters need to be strongly compromized to affect atherosclerotic lesions. The reasons for the discrepant finding to our study are likely multifactorial. In the study by *Porteiro et al*., NTCP inhibition with Bulevirtide nearly doubled plasma bile acid levels compared to controls [39]. In contrast, although plasma bile acids were significantly elevated in our study, even the combined treatment with bulevirtide and rifampicin did not reach the concentrations observed in the bulevirtide–treated group of *Porteiro et al.* This discrepancy may reflect the difference between partial OATP inhibition by rifampicin and the complete deficiency of OATPs by knock-out. In addition, the treatment duration in the study by *Porteiro et al.* (10–12 weeks) was markedly longer than in the present study, raising the possibility that higher levels of bile acids and a greater reduction in lesion size might have been reached with prolonged treatment. *Porteiro et al.* further demonstrated that treatment with bulevirtide reduced plasma cholesterol levels in OATP1a1⁻/⁻ Ldlr⁻/⁻ mice and proposed that the observed decrease in lesion size was primarily mediated by this lipid-lowering effect. In contrast, plasma cholesterol levels in the present study remained unaffected by bulevirtide treatment, which may as well explain the absence of a reduction in atherosclerotic lesion area. It is worth noting that in a human trial, no reduction in plasma cholesterol was observed either. This may be because humans experience a greater increase in bile acids, likely due to less compensation by OATP in bile acid uptake [40]. However, the impact on atherosclerotic lesion remains unclear as studies in FXR-deficient mice or mice models investigating treatment with FXR-agonists showed as well controversial effects on lesion size [20–24].

Beyond overall plaque size, lesion composition is of great relevance, with macrophage content – through their transformation into foam cells and subsequent contribution to necrotic core formation – being crucial for atherosclerotic plaque stability. In this context, the present study provides novel insights by demonstrating that even after only four weeks of treatment, a significant reduction in macrophage content within the lesion area was observed in the combined treatment group (bulevirtide plus rifampicin). As neither bulevirtide nor rifampicin monotherapy showed a significant change in macrophage content within atherosclerotic plaques, a direct effect of either agent alone appears doubtful. The observed reduction in macrophage content in the combined treatment group could therefore be attributed to the markedly elevated bile acid levels, which were greatest under combined therapy. This might be mitigated through the FXR and the TGR5 signaling pathways which have shown anti-inflammatory effects. Previous investigations in mice demonstrated that activation with an TGR5 agonist resulted in a reduction of macrophages in atherosclerotic plaques [18]. Furthermore, bile acids are known to modulate macrophage function through the receptor FXR, which is expressed on blood-derived macrophages [21]. Activation of the FXR pathway by various agonists has been shown to attenuate macrophage cholesterol uptake, foam cell transformation and suppress the induction of proinflammatory mediators [21,41]. Moreover, FXR-null macrophages displayed significantly lower ox-LDL uptake in macrophages derived from FXR-null mice [20]. Besides the direct effect on macrophages, FXR ligands were also able to activate the inhibition of NF-κB–dependent signalling in in-vivo studies of vascular smooth muscle cells, which are as well crucial in macrophage migration [16]. In light of this, the present study broadens the understanding of the anti-inflammatory effects of bile acids by demonstrating that elevated bile acid levels after four weeks of treatment with bulevirtide and rifampicin are associated with lower macrophage content in early atherosclerotic plaques.

This study has limitations that should be considered when interpreting the findings. First, the treatment period of four weeks was relatively short and may not have been sufficient to detect potential effects on overall plaque size. Longer interventions could potentially reveal more pronounced structural changes as shown in existing literature [39]. Additionally, collagen-specific staining on adjacent aortic root sections would have provided further insight into plaque composition and stability. Second, while a significant association between plasma bile acid levels and macrophage content within the plaques was observed, these data are correlative and do not establish a direct causal relationship. Additional mechanistic studies are required to confirm whether bile acids directly modulate macrophage infiltration or survival within atherosclerotic lesions. Furthermore, another limitation of this study is the lack of direct baseline lesion assessment in the same cohort. However, the expected disease stage at treatment initiation is well supported by existing literature [32]. Finally, it is important to consider the potential confounding effect of body weight, as the treatment groups, particularly the combined group and the bulevirtide group, exhibited significantly lower body weights compared to the control group.

## Conclusions

In summary, the present study demonstrates that combined treatment with bulevirtide and rifampicin significantly reduces macrophage content in early atherosclerotic lesions of ApoE-deficient mice, which could be mediated by elevated plasma bile acid levels. While no effect on overall lesion size was observed, the findings underscore the association of bile acids with the modulation of plaque composition and stability. These results provide novel mechanistic insights and warrant further investigation in longer-term studies to clarify the causal role of bile acids in atherosclerosis progression.

## Supporting information

S1 Fig Body Weight. Data are presented as mean ± standard deviation (SD). Control (n = 11), Bulevirtide (n = 13), Rifampicin (n = 15), combination therapy Bulevirtide/Rifampicin (n = 14).
(PDF)
