# Supplementary figures and images for: Effects of bulevirtide on atherosclerosis in an ApoE-deficient mouse model
Source: PLoS One. 2026 May 26;21(5):e0349211. doi: 10.1371/journal.pone.0349211 (PMC13210393; doi:10.1371/journal.pone.0349211)

## Mouse Weight

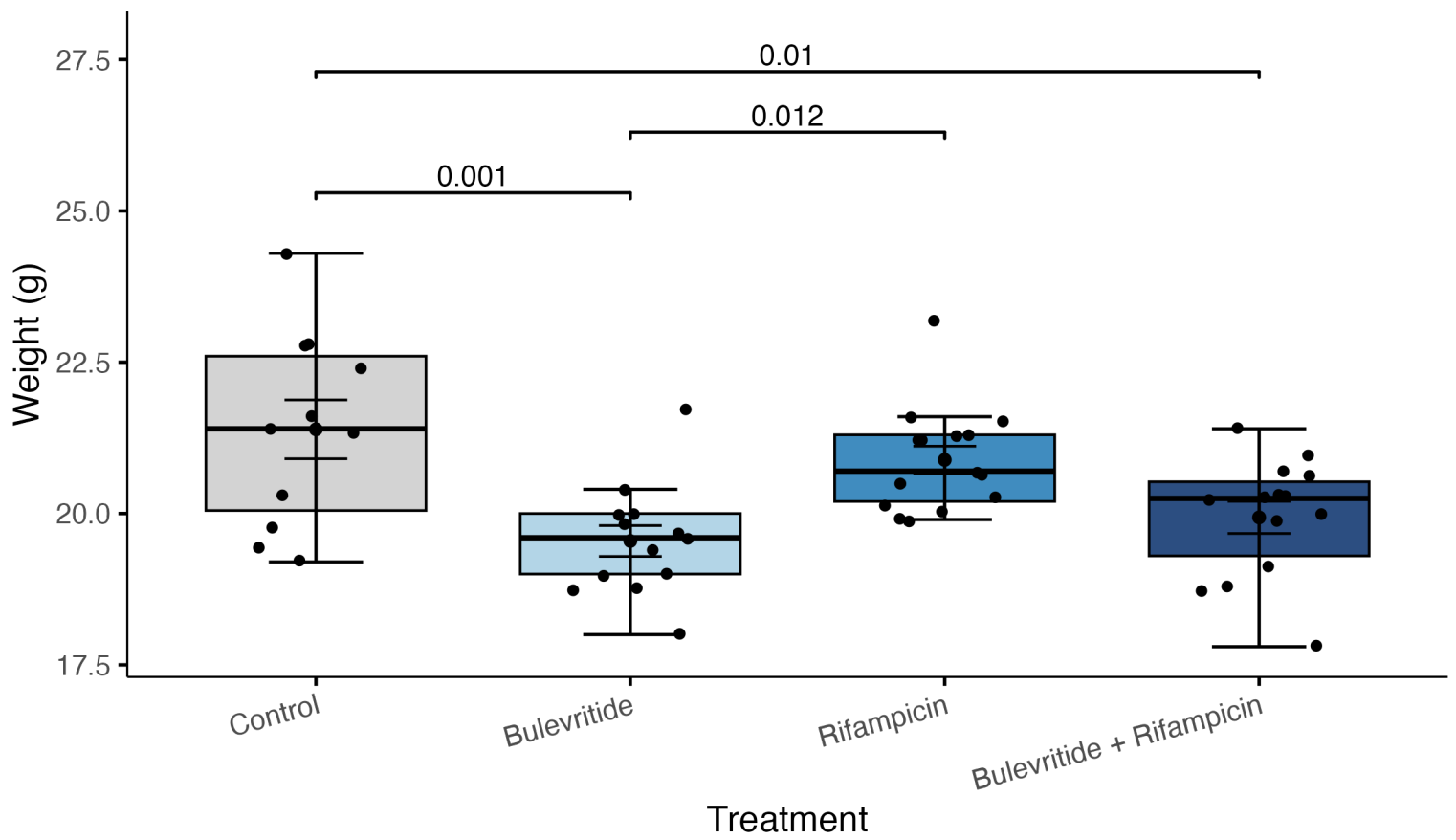

Supplement: S1 Fig — Data are presented as mean ± standard deviation (SD). Control (n = 11), Bulevirtide (n = 13), Rifampicin (n = 15), combination therapy Bulevirtide/Rifampicin (n = 14). (PDF) [file pone.0349211.s001.pdf]
